# Safety and utility of ultrasound-guided superior cervical ganglion block for headaches and orofacial pain: a retrospective, single-center study of 10 patients

**DOI:** 10.1186/s40981-023-00613-z

**Published:** 2023-04-29

**Authors:** Aiko Maeda, Yoji Chikama, Ryudo Tanaka, Masachika Tominaga, Kazuhiro Shirozu, Ken Yamaura

**Affiliations:** 1 Department of Anesthesiology and Critical Care Medicine, Kyushu University Hospital, 3-1-1 Maedashi, Higashi-Ku, Fukuoka City, 812-8582 Japan; 2 Operating Rooms, Kyushu University Hospital, Fukuoka, Japan; 3 Department of Anesthesiology and Critical Care Medicine, Kyushu University Graduate School of Medicine, Fukuoka, Japan

**Keywords:** Superior cervical ganglion block, Headache, Orofacial pain, Stellate ganglion blocks, Ultrasound guidance

## Abstract

**Background:**

Several new ultrasound-guided superior cervical ganglia blocks (U-SCGBs) have been proposed to overcome the shortcomings of conventional superior cervical ganglia blocks; however, their clinical utility and practicality have not yet been demonstrated. The aim of this study was to evaluate the safety and utility of a new method of U-SCGB.

**Methods:**

We retrospectively collected data on patients who underwent U-SCGB for the treatment of headaches and orofacial pain at a single center. U-SCGB was performed by injecting 2–3 mL of 1% mepivacaine posterior to the internal carotid artery, just above the bifurcation. The Wilcoxon signed-rank test was used to compare pain scores. Numerical data are expressed as the mean ± standard error.

**Results:**

The total number of U-SCGB procedures was 43. All procedures were accompanied by Horner’s sign. The numerical rating scale score for pain (possible scores, 0–10) was reduced predominantly from 7.0 ± 0.7 before treatment to 4.5 ± 0.7 at the follow-up (*p* = 0.014).

**Conclusion:**

U-SCGB was considered a clinically useful and accurate treatment for headaches and orofacial pain in this study.

## Background

Headaches and orofacial pain may be caused by abnormalities in autonomic innervation and/or function. The cervical sympathetic ganglia consist of the superior, middle, intermediate, and inferior cervical sympathetic ganglia. Stellate ganglion block (SGB) refers to the blocking of the sympathetic chains/ganglia of the cervical and upper thoracic regions and is utilized to diagnose and treat pain-related diseases of the head, neck, mouth, face, and upper extremities [1]. Although traditional SGB has given way to a safer, ultrasound-guided approach, it can still be difficult to avoid puncturing the arteries due to the puncture level of C6–7. This poses a risk of vertebral or thyroid arterial puncture, which may cause local anesthetic intoxication and fatal retropharyngeal hematoma with or without ultrasound guidance [3].

The superior cervical ganglia (SCG) are cervical sympathetic ganglia located cephalad to the common carotid artery bifurcation. Although the effectiveness of SCG blocking alone has been reported in the cervical sympathetic nerve for trigeminal neuralgia, postherpetic neuralgia, and chronic facial pain [6], conventional landmark techniques for SCG block are not widely used because of the risks of infection or accidental injection into the internal carotid artery [7]. Several anatomical and morphological studies have proposed new ultrasound-guided SCG blocks to solve these problems; however, their clinical utility and practicality have not yet been demonstrated [10]. In this study, we aimed to propose a method for the performance of SCG blocks via ultrasound guidance as suggested in the above reports and verify whether ultrasound-guided SCG block is useful for the treatment of headaches and orofacial pain.

## Methods

We conducted a retrospective cohort study by reviewing the clinical records of patients diagnosed with headache and orofacial pain who underwent an ultrasound-guided SCG block at the pain management unit of Kyushu University Hospital. This study was approved by the Kyushu University Hospital Ethics Committee on August 30, 2022 (ID number 2106901) and performed in accordance with the 1964 Helsinki Declaration and its later amendments. Due to the retrospective design of this study, the requirement for written consent was waived for treated patients unless they refused. Moreover, an opt-out approach was implemented by publishing the information explaining the study on our website and guaranteeing the opportunity to refuse the use of information whenever possible until March 31, 2023 (address of the website: https://www.kuaccm.med.kyushu-u.ac.jp).

Each treated patient enrolled in this study was individually informed in advance using sentences, and an anatomical model of the cervical region that ultrasound-guided SCG block would be performed in the following methods, and informed consent was obtained in writing.

### Participants and methods

We collected data on patients who underwent ultrasound-guided SCG block from January 2019 to May 2022. Patients who were diagnosed with headaches and orofacial pain refractory to conservative treatment for at least 3 months were included in this study. The diagnoses of these patients were based on the International Classification of Headache Disorders of the International Headache Society [13]. Patients with incomplete diagnoses were excluded from the study. Ten patients conformed to our criteria, and the following data were collected from their hospital medical records: age, sex, diagnosis, number of blocks, signs of Horner’s syndrome, transition of pain score, analgesic dose, and the number of adverse events.

### Ultrasound-guided superior cervical ganglion block procedure and clinical assessments

**Fig. 1:**
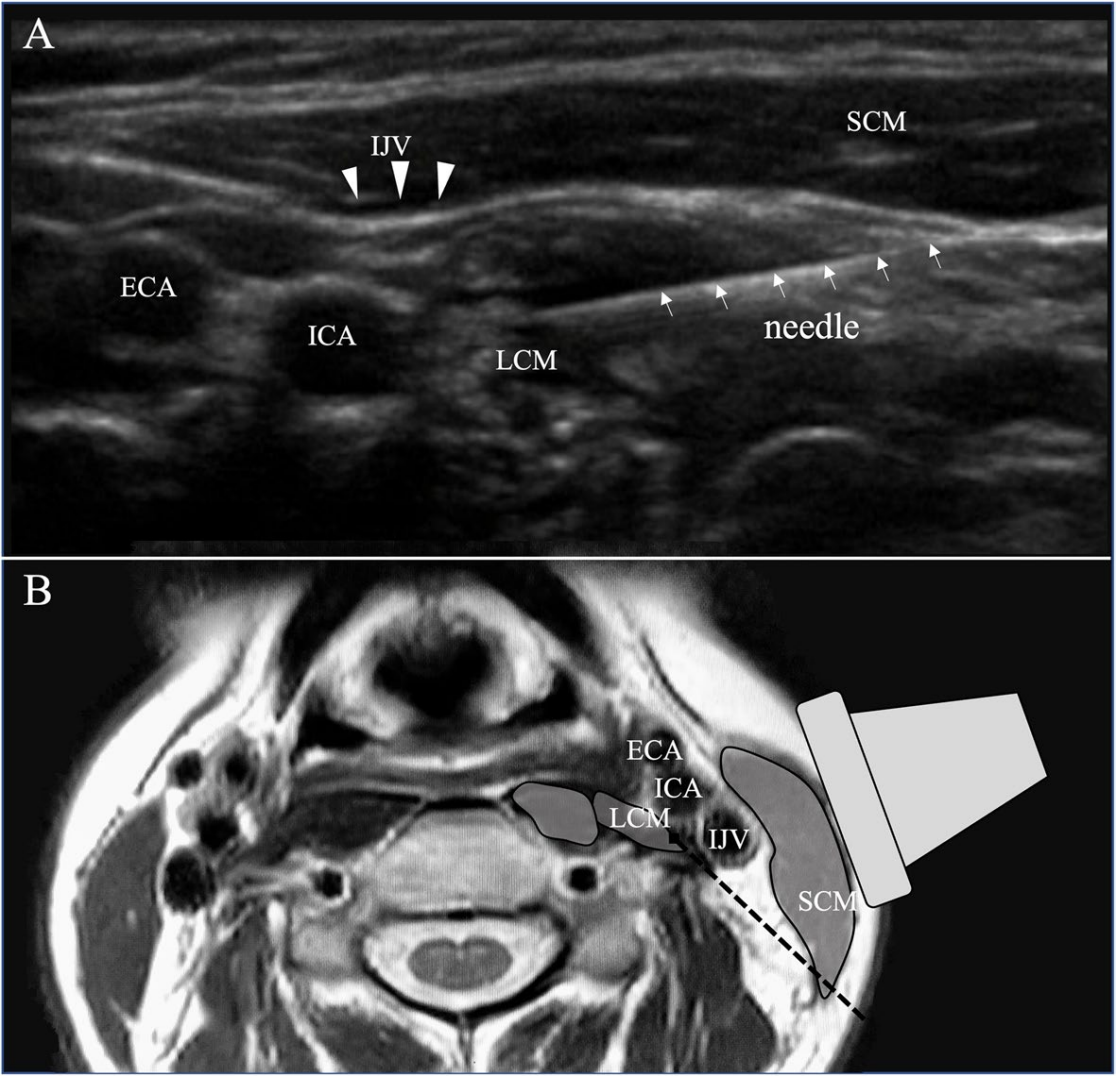
A represented ultrasound image of the left common carotid bifurcation (**A**) and axial section of T2 weighted magnetic resonance image at approximately the same level (**B**). **A** A needle (white arrow) is visualized from the right side of the image toward the LCM. The hypoechoic image in the LCM is due to local anesthetic injection. **B** The black dashed line indicates the expected route of needle insertion. ICA, internal carotid artery; ECA, external carotid artery; IJV, internal jugular vein (white arrowheads); LCM, longus capitis muscle; SCM, sternocleidomastoid muscle

The patients were placed in the lateral decubitus position with the affected side up, following which the mastoid process, hairline, and neck were disinfected. The common carotid artery was identified via ultrasound; a high-resolution linear probe (SonoSite SII; Fujifilm, Tokyo, Japan) was positioned vertically to the blood vessel. The probe was gradually moved in the cranial direction to the position of the carotid bifurcation. A representative ultrasound image is presented in Fig. 1. A needle (25-G injection needle, 60 mm; Top Corporation, Tokyo, Japan) was introduced using the in-plane approach. The C3 vertebral transverse process, vertebral artery, longus capitis muscle (LCM), and sternocleidomastoid muscle have been located to determine the puncture route, and 2–3 mL of local anesthetic (1% mepivacaine) was injected just behind the internal carotid artery. During the ultrasound-guided SCG block procedure, the patients were monitored for vital signs (blood pressure every 10 min and continuous pulse rate and SPO_2_) for approximately 30 min. The reduction in pain and signs of Horner’s syndrome were examined as the primary endpoints, and the secondary endpoint was the number of adverse events after the procedures. The patient was evaluated for signs of Horner’s syndrome 15–30 min after each ultrasound-guided SCG block. The level of pain was graded on a numerical rating scale (NRS) ranging from 0 to 10, 3 months after the start of treatment. On the day of evaluation, the physician asked the patient about the maximum and minimum NRS scores for the past few days; this information was noted in the patient’s medical record, and these scores were extracted.

### Statistical analysis

All values are expressed as the mean ± standard error. Statistical analysis was performed using the Wilcoxon signed-rank test for changes in NRS scores before and after the completion of the nerve blocks. Statistical significance was set at *p* < 0.05.

## Results

This study consisted of 43 ultrasound-guided SCG block procedures (the average number of blocks was 4.3 ± 0.4) performed on 10 patients, with a female predominance (2 male and 8 female patients). The patient’s diagnoses were persistent idiopathic facial pain (PIFP), occipital neuralgia, painful post-traumatic trigeminal neuropathy, burning mouth syndrome, and postherpetic trigeminal neuropathy. The average age of the patients was 51.3 ± 4.5 (range, 32–69) years. The disease period, namely the time from disease onset to ultrasound-guided SCG block, was 21.6 ± 5.7 (range, 3–60) months. Signs of Horner’s syndrome were observed after all 43 procedures (100%). The maximum and minimum NRS were significantly reduced after 3 months of the first ultrasound-guided SCG block, from 7.0 ± 0.7 to 4.5 ± 0.7 (*p* = 0.014) and from 3.4 ± 0.7 to 1.4 ± 0.4 (*p* = 0.018), respectively. Table 1 summarizes the diagnosis, disease duration, and number of ultrasound-guided SCG blocks of each case, and the changes in maximum NRS and analgesic consumption. In six cases, there was no change in analgesic consumption before or after the procedure; in three cases, the dose was reduced, and in one case, the analgesics were increased. Nine patients were able to discontinue ultrasound-guided SCG block within 3 months, and one patient continued treatment once a month. None of the patients in this study developed significant changes in vital signs associated with ultrasound-guided SCG blocks. No adverse events were observed after the 43 ultrasound-guided SCG block procedures performed in this study.

**Table 1:** Diagnosis, disease duration, and number of ultrasound-guided SCG blocks of each case and changes in maximum NRS and analgesic consumption

	**Sex/age**	**Diagnosis**	**Disease duration (months)**	**Number of U-SCGB in 3 months**	**Maximum NRS before U-SCGB**	**Maximum NRS three months after starting U-SCGB**	**Analgesics per day before U-SCGB**	**Analgesics per day 3 months after starting U-SCGB**	**Increase/decrease of analgesics**
1	F/62	PIFP	36	3	5	7	None	None	No change
2	F/32	PIFP	18	6	8	5	Loxoprofen (120 mg), acetaminophen (3600 mg), pregabalin (50 mg)	Acetaminophen (1200 mg)	Decrease
3	F/34	Occipital neuralgia	6	3	4	1	Loxoprofen (60 mg)	None	Decrease
4	F/35	PPTTN	4	4	4	1	None	None	No change
5	F/45	Occipital neuralgia	14	5	9	5	Carbamazepine (200 mg)	None	Decrease
6	F/65	Burning mouth syndrome	60	6	9	5	Mirogabalin (20 mg), gabapentin (300 mg)	Mirogabalin (20 mg), gabapentin (300 mg)	No change
7	F/69	PHTN	3	4	8	3	Pregabalin (75 mg)	Pregabalin (150 mg)	Increase
8	M/52	PPTTN	15	5	6	4	Mirogabalin (20 mg)	Mirogabalin (20 mg)	No change
9	M/65	Burning mouth syndrome	24	3	8	8	None	None	No change
10	F/54	PHTN	36	4	9	6	Mirogabalin (5 mg)	Mirogabalin (5 mg)	No change

*SCG* superior cervical ganglia, *NRS* numerical rating scale, *U-SCGB* ultrasound-guided superior cervical ganglion block, *PIFP* persistent idiopathic facial pain, *PPTTN* painful post-traumatic trigeminal neuropathy, *PHTN* postherpetic trigeminal neuropathy

## Discussion

Since all preganglionic fibers of the cervical sympathetic ganglia originate in the thoracic spinal cord and ascend to the sympathetic chains/ganglia, blocking the lower cervical sympathetic ganglia may block SCG. However, in the treatment of head and neck pain, the SCG blockade alone is sufficient. SCGs, the largest of the cervical sympathetic ganglia, are spindle-shaped tissues, 10–30 mm in length [11]. Previous reports have indicated that SCGs communicate not only with the various brain nerves but also with branches of the C1–4 cervical nerves [10]. After leaving the SCG, the postganglionic sympathetic fibers are distributed to the head and neck muscles, carotid bifurcation, sympathetic ganglion of the salivary gland, common carotid artery, and internal jugular vein, along the brain nerves and C1–4 afferent nerves. Given these morphological properties, blockade of the SCG may be an effective treatment for headaches and orofacial pain [11].

According to cadaveric studies [10], SCGs are located posterior to the carotid bifurcation, anterior to the LCM in front of the second to third cervical transverse processes, and slightly below the mandibular angle, with almost no interindividual differences. Ultrasound was used under the mandible with the cadaver in the supine position in the abovementioned studies. Those procedures were not clinically practical, as the mandible is an obstacle to the rapid and accurate execution of complex procedures under ultrasound guidance. In this study, the needle was inserted posterior to the linear probe with the patient in a lateral decubitus position for ease of access below the mandible. This method has the advantage that local anesthetic can be easily injected just posterior to the internal carotid artery. Furthermore, we believe that this would be a safer procedure because there are no organs or major blood vessels in that area. However, since the needle advances toward the carotid artery, its tip must be unambiguously confirmed ultrasonically using the in-plane technique. The observation of Horner’s signs following every ultrasound-guided SCG block, even with a small amount of local anesthetic, was consistent with previous studies showing little anatomical variability in SCG location. We believe our method is an accurate approach to ultrasound-guided SCG block. The results of this study suggest that ultrasound-guided SCG block is highly effective for headache and orofacial pain, as maximum and minimum pain scores after treatment were significantly reduced compared to those before treatment, despite the fact that many patients decrease or do not change their analgesics. Some headaches and orofacial pain, such as PIFP and burning mouth syndrome, have been previously reported to be female-dominant [14]. Therefore, although the number of patients in this study was small, it was considered appropriate as a representative patient group for headaches and orofacial pain.

Any part of the blockage of the cervical sympathetic chains/ganglia could potentially block the vagus or phrenic nerves. More specifically, the vagus nerve runs between the internal jugular vein and the vertebral artery and near the cervical sympathetic chain/ganglion and might be blocked if the cervical sympathetic chain were blocked. However, the circulatory system is balanced by autonomic homeostasis, suggesting that unilateral SGB performed clinically will not significantly disturb the circulation balance [15]. Moreover, the SCGs are located close to the cervical plexus [10]. SCG block in patients with cervical spinal cord injury and other phrenic nerve-dependent breathing is considered dangerous, as is C6-level SGB and brachial plexus block with an interscalene approach. Although we observed no changes in blood pressure, pulse, or SPO_2_ during ultrasound-guided SCG block, we did not monitor whether the vagus and phrenic nerves were blocked in this study. Given the small sample size of this study, complications of ultrasound-guided SCG block should be further investigated.

This study has several limitations. First, we included only patients with pain refractory to conservative treatment and who were treated with ultrasound-guided SCG block, with no control group. Thus, the degree of superiority of the ultrasound-guided SCG block procedure compared with other treatments or the natural course of the disease with conservative therapy is unknown. Second, as with other chronic pain, psychosocial considerations are important in the management of headaches and orofacial pain [16]; however, such evaluations were not performed in this study. Third, as this was a retrospective study and the amount of oral medicine was increased or decreased at the discretion of each doctor, it was not possible to determine the effect of ultrasound-guided SCG block under specific conditions. Finally, the sample was very small (*n* = 10); larger studies are required to verify the utility of our procedure for the treatment of headaches and orofacial pain.

## Conclusions

The SCG can be reliably blocked via ultrasound-guided injection of a small amount of local anesthetic posterior to the internal carotid artery. Our results suggest that ultrasound-guided SCG block is a promising alternative to conventional SGB for the treatment of sympathetically maintained headache and orofacial pain, as the latter may cause fatal complications such as retropharyngeal hematoma.

## Data Availability

Not applicable.

## Abbreviations

LCM: Longus capitis muscle
NRS: Numerical rating scale
PIFP: Persistent idiopathic facial pain
SCG: Superior cervical ganglia
SGB: Stellate ganglion blocks
